# Retraction: MicroRNA-371-5p targets SOX2 in gastric cancer

**DOI:** 10.18632/oncotarget.26037

**Published:** 2018-08-21

**Authors:** Yu-Ji Li, Ming Dong, Fan-Min Kong, Jian-Ping Zhou, Dong Liang, Huan-Zhou Xue

**Affiliations:** ^1^ Department of General Surgery, The First Affiliated Hospital, China Medical University, Shenyang 110001, Liaoning, P.R. China

**This article has been retracted:** From the The First Hospital of China Medical University Professor Committee, in response to the Journal’s request:

The First Hospital of China Medical University Professor Committee convened the 14th plenary meeting to investigate Yu-Ji Li’s paper published in Oncotarget (referred to as ‘the Journal’ in the rest of the letter), in response to the Journal’s editorial board’s request. A thorough investigation and open discussion was held in the meeting that included all department members in charge of scientific affairs.

At the meeting, Dr. Yu-Ji Li reported all events in the production of his paper. He admitted the problems in the publication that were identified by the Journal and acknowledged that he was responsible for the incidents. The chairman of Dr. Li’s department also detailed on the incidents and pointed out the loopholes in the paper. Inquiries were made and documents were carefully checked by the Committee members. The Professor Committee agreed that Yu-Ji Li was negligent in his research attitude and was unable to offer complete original materials. After the discussion, the decision was made as the following:

1) Due to repeated and manipulated use of figures that misled the scientific conclusion, we ask for retraction of the paper by Dr. Yu-Ji Li from the Journal. (Dr. Li himself previously attempted to retract but his plea was unclear in expression).

2) Apologies must be issued to the Journal and its readerships for the authors’ negligence and lack of rigorous academic integrity.

3) The University will take strict measures against the misconduct by the authors of the paper according to relevant institutional regulations.

Original article: Oncotarget. 2016; 7:31993-32005. https://doi.org/10.18632/oncotarget.8289

